# Systemic administration of c-Kit^+^ cells diminished pulmonary and vascular inflammation in rat model of chronic asthma

**DOI:** 10.1186/s12860-022-00410-z

**Published:** 2022-02-24

**Authors:** Sajjad Taghizadeh, Rana Keyhanmanesh, Reza Rahbarghazi, Jafar Rezaie, Aref Delkhosh, Mehdi Hassanpour, Hossein Heiran, Arshad Ghaffari-Nasab, Mahdi Ahmadi

**Affiliations:** 1 Department of Physiology, Faculty of Medicine, Tabriz University of Medical Sciences, Tabriz, Iran; 2 Student Research Committee, Tabriz University of Medical Sciences, Tabriz, Iran; 3 Drug Applied Research Center, Tabriz University of Medical Sciences, Tabriz, Iran; 4 Stem Cell Research Center, Tabriz University of Medical Sciences, Tabriz, Iran; 5 Department of Applied Cell Sciences, Faculty of Advanced Medical Sciences, Tabriz University of Medical Sciences, Tabriz, Iran; 6 Solid Tumor Research Center, Cellular and Molecular Medicine Institute, Urmia University of Medical Sciences, Urmia, Iran; 7 Department of Clinical Biochemistry and Laboratory Medicine, Tabriz University of Medical Sciences, Tabriz, Iran

**Keywords:** Chronic Asthma, C-Kit cells, Rat, Pulmonary and Vascular inflammation, Chronic model

## Abstract

**Background:**

To circumvent some pitfalls related to acute status, chronic model of asthma is conceived to be more suitable approach to guarantee the conditions which are similar to human pulmonary disease. Here, possible therapeutic mechanisms were monitored by which c-kit^+^ bone marrow cells can attenuate vascular inflammation in rat model of chronic asthma.

**Results:**

Data revealed c-Kit^+^ cells could significantly reduce pathological injures in asthmatic rats via modulating the expression of *IL-4, INF-γ*, *ICAM*-1 and *VCAM-1* in lung tissues and TNF-α, IL-1β and NO levels in BALF (*p* < 0.001 to *p* < 0.05). Besides, c-Kit^+^ cells reduced increased levels of VCAM-1 evaluated by immunohistochemistry staining. In contrast to c-Kit^+^ cells, c-Kit^−^ cells could not exert beneficial effects in the asthmatic conditions.

**Conclusion:**

Overall, we found that systemic administration of C-kit positive cells can diminish pulmonary and vascular inflammation of chronic asthmatic changes in a rat model. These cells are eligible to suppress inflammation and nitrosative stress in lung tissue coincides with the reduction of pathological changes. These data indicate that C-kit positive cells be used as an alternative cell source for the amelioration of asthmatic changes.

## Background

The occurrence of asthma can lead to debilitating pathological condition of lung tissue which is mainly characterized by prominent airway conduit inflammation. These conditions affect over 400 million individuals globally with limited therapeutic options [1, 2]. Chronic structural changes in lung architecture of asthmatic subjects such as airways hyper responsiveness and intermittent bronchoalveolar constriction are thought to accompany particularly by Th2-driven inflammatory responses, contributing to asthma symptoms [3, 4]. Due to ethical issues, investigating underlying mechanisms in asthmatic conditions are not applicable in the clinical setting. In this regard, animal models are an alternative for the evaluation of different medications and therapeutic approaches [2]. To date, both acute and chronic asthma animal models can be used by application of certain allergens like ovalbumin [3]. In the acute conditions, the inflammation intensity and stability of pathological remodeling are not permanent and hence they could not be completely applicable to the human asthmatic niche [3, 5, 6]. In contrast, repetitive allergen provocation in the chronic model of asthma is touted to be suitable method to mimic the conditions which are similar to human pulmonary disease. For instance, the continuous use of allergens in small quantitative are enough to yield typical asthmatic features [3, 5]. Regarding the entity of asthma and complexity of pathological pattern, it seems that the regeneration of injured and inflamed pulmonary tissue via conventional medications is not completely feasible [7–9]. In this context, Most efforts have been targeted at the discovery of de novo therapeutic agents in asthmatic patients from the past to the present time [2, 7, 8, 10]. As a correlate, cell-based therapy is touted as one of available strategies with potential to restore normal function of pulmonary tissue after the asthmatic changes [8, 11]. Among several cell types, stem cells have provided hopeful avenues for the acceleration of healing procedure in asthmatic injuries [8, 10]. It has been shown that stem cells from different origins could restore the function of injured pulmonary cells after transplantation. Notably, the type of stem cells, administration route, injection volume, and initial number of transplant cells could affect the regeneration outcome [2, 7, 8, 12]. However, typical and optimum values are lacking and subject of interest. Among multiple sets of stem cells and progenitors have been applied for different diseases, c-Kit positive cells are not routinely administrated for chronic asthma.

The recent experiment conducted by our group revealed the potency of c-Kit^+^ cells in alleviating pathologies and restoration of normal immune cell function associated with the acute asthma in rat model generated by common protocol (32-day allergen exposure time) [9, 12]. Whether the transplant cells can restore inflamed lungs function during the chronic asthma condition is lacking. Along with these comments, we tried to investigate the effect of systemic transplantation of c-Kit^+^ cells isolated from bone medullary in the alleviation of chronic asthmatic in rat model.

## Material

### Animal issues

This is study was done in accordance with the previously published principles (NIH publication no. 85–23, revised 1996) and approved by Local Ethics Committee of Tabriz University of Medical Sciences (No: IR.TBZMED.VCR.REC.1399.078).

#### Establishment of chronic asthma in the rat model

Eight to ten week-old male Wistar rats (*n* = 40) were obtained from our institute animal husbandry. Standard laboratory cages were used (with four rat per/cage). Animals were housed in climate-controlled rooms (20–22 °C) with 55 ± 5% relative humidity) and standard light/dark cycle. To feed animals, chew diet was used with free access to water. Next, 8 rats were blindly selected and marrow-derived c-kit^+^ and c-kit^−^ cells were isolated, and the rest of rats were randomly subjected to four experimental groups (*n* = 8) as follows; Control rats (C group); rats exposed to ovalbumin for 70 days (Chronic asthma: CA group); In the CA rats, 50 μl cell-free PBS was intravenously injected. In CA + c-Kit^−^ and CA + c-Kit^+^ groups, a single dose of 3 × 10^5^ c-Kit^−^ and 3 × 10^5^ c-Kit^+^ cells was used respectively.

Here, we used a 70-day sensitization procedure according to previously published protocols with some modifications [13]. In the CA rats, each animal was sensitized with the combination of OVA (1 mg/ml; Sigma-Aldrich) and aluminum hydroxide (200 µg/ml) injected intraperitoneally on days 0 and 7. Every two days from days 14 to 70, the rats were placed in a Plexiglas chamber (approximately 30 cm × 20 cm × 20 cm in volume) connected to a nebulizer (CX3, Omron Co., Netherland) and subjected to OVA (1%) inhalation for 30 min. Rats in the control group were exposed to PBS using the same protocol.

**Fig. 1:**
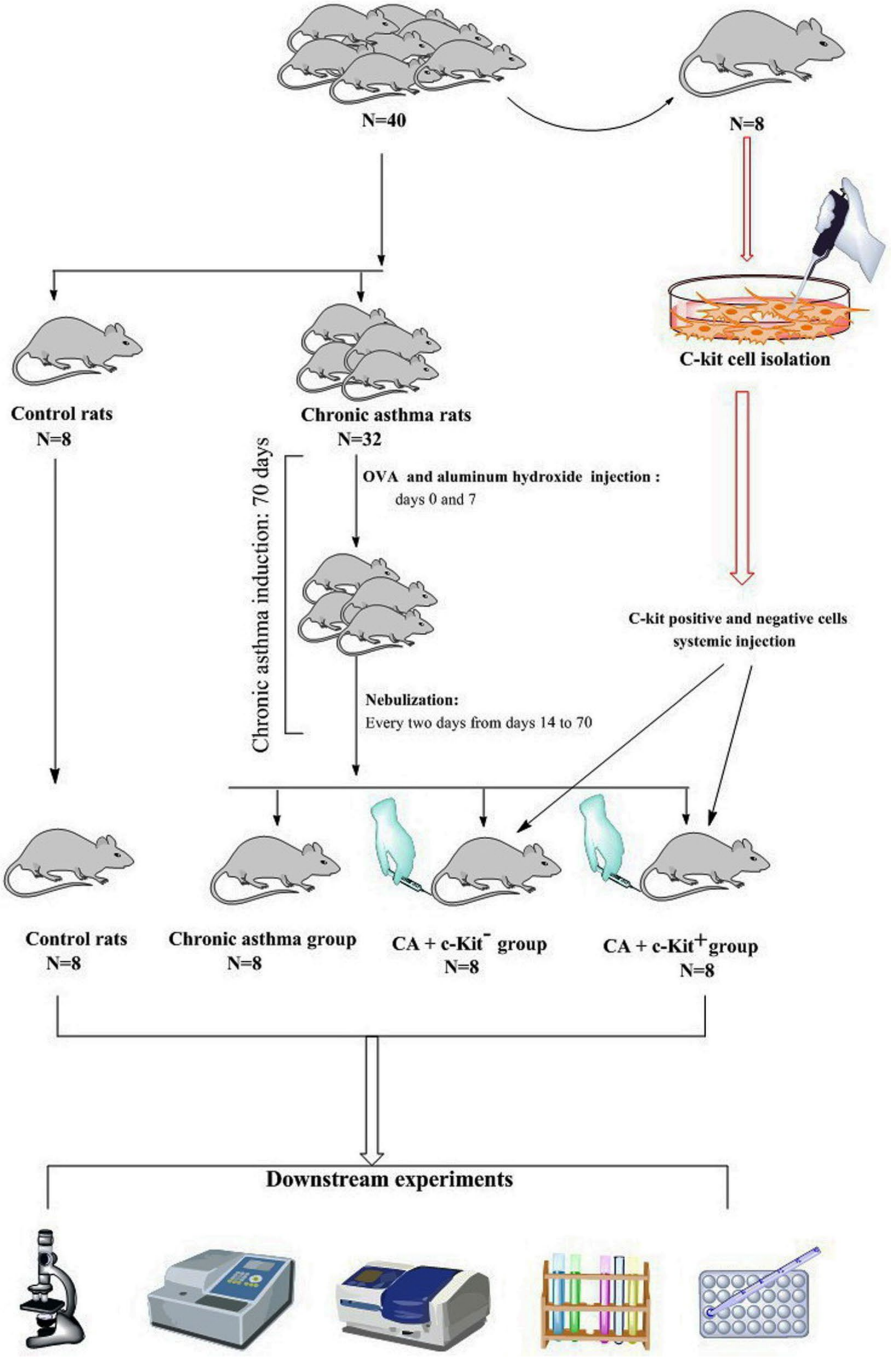
Brief description of experimental procedure in this study

Once the asthma is induced, the animals were deeply anesthetized by administrating Ketamine (i.p.; 75 mg/kg) and Xylazine (i.p.; 3 mg/kg). Before surgical operation, pedal withdrawal reflex to pinch was monitored. 50 μl PBS with re-suspended c-Kit^−^ and c-Kit^+^ cells was injected via tail vein (Ahmadiet al., 2017; Ramachandranet al., 2015). In the C and CA groups, 50 μl PBS-free cells were injected via the same route. Rats of all groups were euthanized 14 days post injection (Fig. 1) [2, 9].

### c-Kit cells enrichment

Eight rats without any manipulations were killed using surplus dose of Ketamine and Xylazine. The femurs were excised and medullary content extracted after extremities removal. The marrow cells were triturated using PBS solution enriched with 2% fetal bovine serum (FBS; Gibco). Mononuclear cells (MNCs) were isolated by gradient centrifugation using Ficoll-Paque® (Sigma-Aldrich). The samples were diluted 1: 1 in PBS. An equal volume of Ficoll was overlaid gently to diluted samples and centrifuged at 400X*g* for 20 min. MNCs at the interphase were collected followed by twice PBS washes (each for 5 min). Cells were blocked using PBS with 1% FBS by maintaining at 4˚C for 30 min. After removing supernatant, anti-c-Kit microbeads (Cat no: 130–091-224; Miltenyi Biotec) was used as recommended by manufacturer followed by passing the cells through the LS columns (Miltenyi Biotec). The procedure was conducted according to our previously published protocol [14]. Either c-Kit^+^ or c-Kit^−^ were isolated and transferred into separate tubes and subjected to different assays.

### Cell labeling

Before the injection, both positive and negative c-Kit cells were tracked by incubation with Cell Tracker™ CM-Dil [9]. For this purpose, cells were exposed to Cell Tracker™ CM-Dil (20 µM) inside incubator for 20 min followed by twice with PBS washes. In this study, about 3 × 10^5^ either c-Kit^+^ or c-Kit^−^ cells were administrated via the systemic route.

### Histopathological examination

The right lungs were with Hematoxylin and Eosin (H & E) solution. To show the deposition of collagen fibers and chronic changes, we also performed Masson’s trichrome staining. All typical pathologies related to the asthmatic changes were monitored and compared to the control samples. In order to make a semi-quantitative scale, we used classification as follows; null (0), mild (1), moderate (2), and severe (3).

### Immunohistochemistry (IHC) analysis of VCAM-1

To evaluate whether the administration of C-kit^+^ and/or C-kit^−^ cells in asthmatic rats can alter VCAM-1 levels, we performed IHC analysis according to the previous studies. To this end, the 5 μm-thick slides were deparaffinized and incubated with 3% oxygen peroxide to neutralize endonuclease activity. The slides were incubated with anti-VCAM-1 antibody for 1 h and washed three times with PBS, followed by the addition of HRP conjugated secondary antibody for 1 h. In this study, 3, 3′-Diaminobenzidine (DAB) was used as chromogen.

### Real-time PCR

Here, the transcription of genes like *IL-4, INF-γ*, ICAM-1 and *VCAM-1* was measured. To this end, left lungs were sampled and total RNA content was extracted by RNA extraction mini kit (Cat No: YT9065; Yekta Tajhiz; Iran). Quantity and purity of samples were monitored by a NanoDrop ND-1000 spectrophotometer followed by reverse-transcription into cDNA. PCR reaction was performed using SYBR Green master mix (Cat No: YT2551; YektaTajhiz, Iran) and a Corbett Rotor-Gene 3000 apparatus*.* CT value of each gene was compared to house-keeping gene (*β-actin*) CT using 2^−ΔΔCt^ formula (Table 1).

**Table 1:** Primer list used in this study

Gene	Primer sequence (*5*’-*3*’)	
	**Forward**	**Reverse**
**IL-4**	TAC GGC AAC AAG GAA CAC CAC	CAG ACC GCT GAC ACC TCT AC
**IFN-γ**	GATCCAGCACAAAGCTGTCA	GACTCCTTTTCCGCTTCCTT
**ICAM-1**	TGG AGG TGA CTG AGA AGT TGG	CAC AGT TAC TTG GTC CCC TTC
**VCAM-1**	GTG TGT GAA GGA GTG AAT CTG G	CCA ACA GCA GCA CAT GTC AGA A
**β-actin**	TCCCTGGAGAAGAGCTACG	GTAGTTTCGTGGATGCCACA

### ELISA

TNF-α and IL-1β contents were monitored in BALF in different groups. To collect the BALF, we instilled 1 ml normal saline for 5 time via a catheter connected to trachea. The diluted BALF were centrifuged to exclude the epithelial and immune cells. Thereafter, the content of TNF-α and IL-1β were measured using ELISA [15].

### NO assay

We also measured to levels of NO to monitor nitrosative status using Griess method as described previously by our group [15]. The levels were expressed as nM by reading the OD at 540 nm using a microplate reader and compared to the control levels.

#### Statistical analysis

All quantitative data are shown in means ± SEM. Using a one-way ANOVA with Tukey–Kramer post hoc test, we performed statistical analysis. In order to achieve semi-quantitative data in histological assay, Kruskal–Wallis and Mann–Whitney U tests were used. P values below 0.05 were touted statistically significant.

## Results

### IF imaging revealed the recruitment of c-kit^−^/^+^ cells into the lung parenchyma

**Fig. 2:**
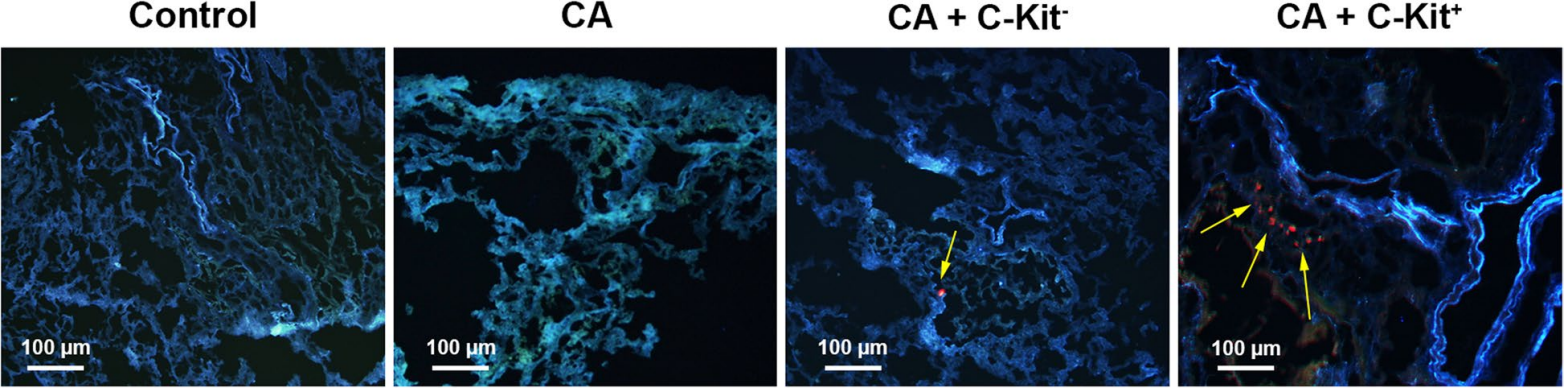
Dil-tagged c-kit^+^ and c-kit^−^ cells can be detected inside pulmonary parenchyma. Data displayed localization of labelled red-colored cells. DAPI (blue color) was used for the staining of cell nuclei. Control rats (C group), sensitized rats (CA group), sensitized rats received c-kit^−^ cells (CA + c-kit^−^ group), sensitized rats received c-kit^+^ cells (CA + c-kit^+^ group)

IF imaging displayed homing of both c-Kit^−^ and c-Kit^+^ cells 14 days after systemic injection. Under microscopic evaluation, we noted the existence of red-colored Dil tagged c-Kit cells adjacent to the local pneumocytes. These cells are distributed randomly inside the pulmonary tissue, indicating the suitability of our protocol in the introduction of these cells into the target sites (Fig. 2).

### C-Kit^+^ cells are eligible to attenuate pathological remodeling during asthmatic changes

**Fig. 3:**
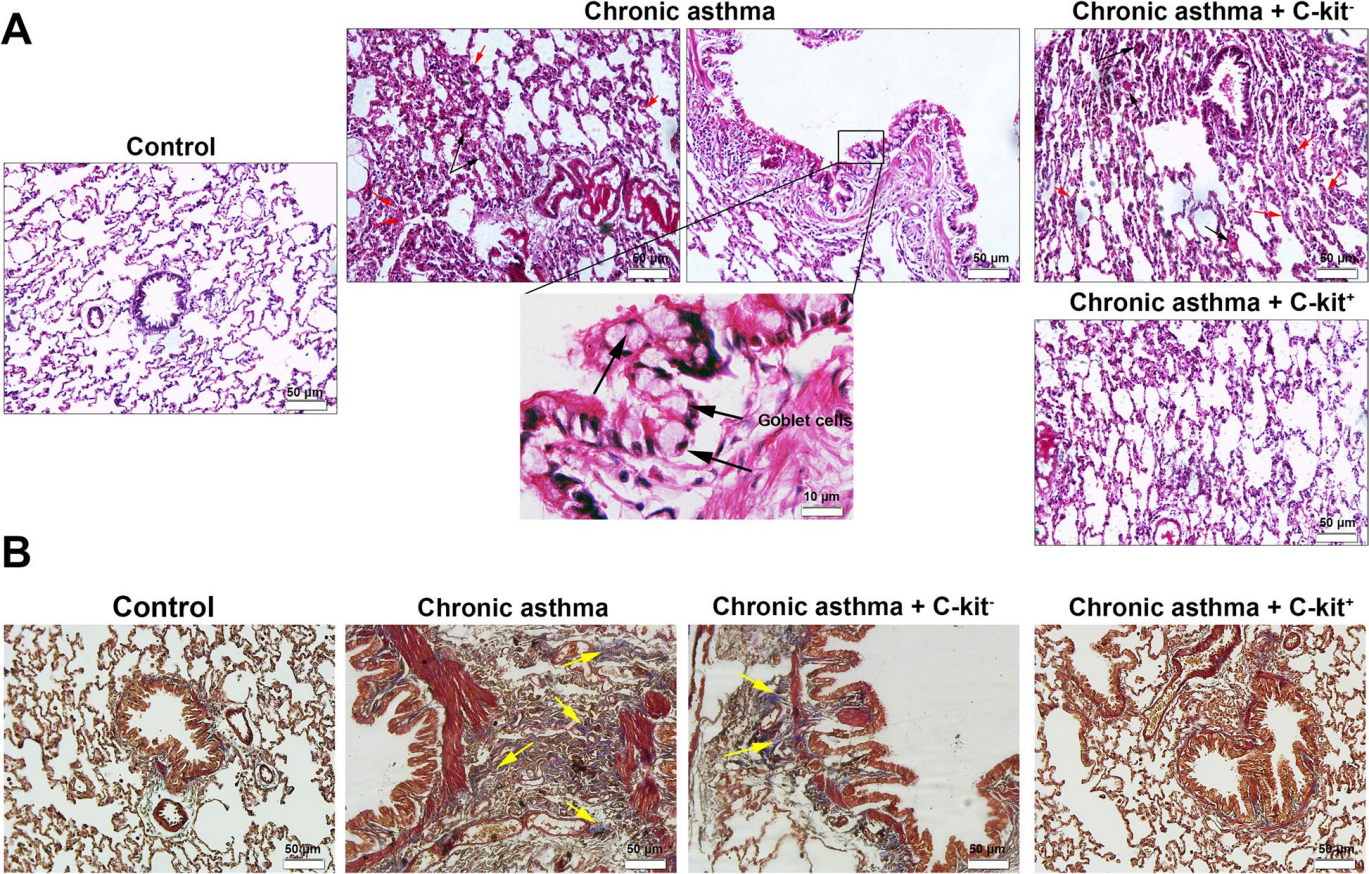
H&E staining. Prominent interstitial pneumonia (arrows), focal haemorrhagia (arrows ‘heads), and emphysema are shown in bright-field imaging. The pathological remodeling coincided with the peri-bronchiolar cuffing, muscular hypertrophy and BALT hyperplasia

**Fig. 4:**
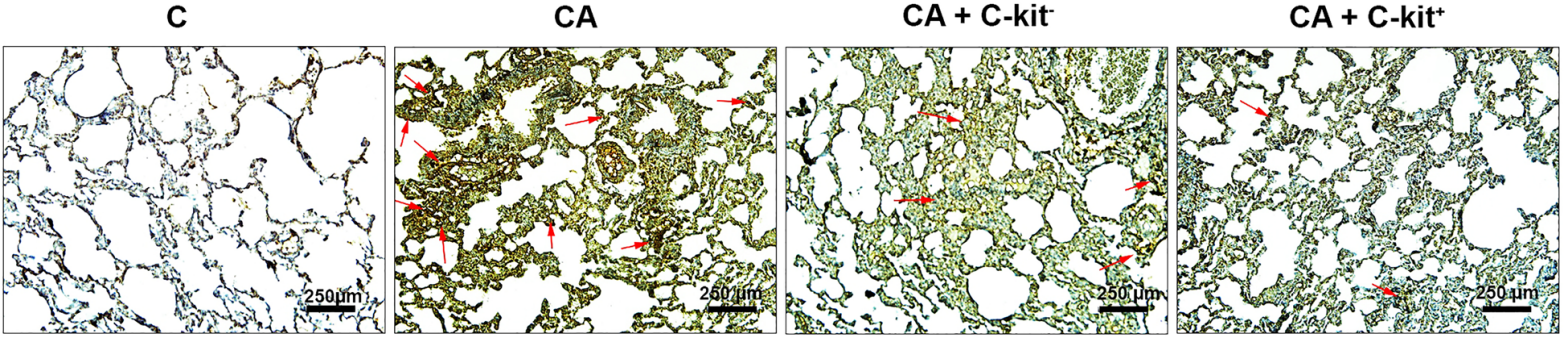
Protein levels of VCAM-1 evaluated using IHC. Data showed that asthma induction promoted VCAM-1 levels compared to the Control group. Administration of C-kit^−^ cells and C-kit^+^ cells can reduce protein levels of VCAM-1. These effects are more prominent in asthmatic group received C-kit positive cells

H & E examination displayed that chronic asthma was successfully induced in rats (Fig. 3, Table 2). Histological values were significantly higher in the lung tissues of all sensitized groups compared to the C group (*p* < 0.001 to *p* < 0.05). These features were indicated by prominent interstitial pneumonia and bulk recruitment of immune cells into the lung parenchyma. Beside, fibrinous exudate with local hyperemia can be detected in asthmatic lungs. Also, we noted hyperplasia of goblet cells and massive bronchiolar epithelial cells injury in which a large number of epithelial cells were detached and slaughtered into the lumen of bronchiolar airway. The goblet cell hyperplasia coincided with an increased mucus production, showing the chronic changes inside the lung parenchyma. Bright-filed imaging showed the significant injury of brush borders and the detachment of epithelial layer from the underlying membrane. The chronic changes led to thickening of alveolar sac wall which can decrease the gas interchange between the blood and air sacs. According to our data, we found that systemic injection of c-kit^+^ cells can decrease the intensity and chronic inflammatory responses inside the lungs tissue compared to the asthmatic group but these values were unchanged in the rats received c-kit^−^ cells (Fig. 3, Table 2). To exactly monitor the changes in collagen deposition related to chronic pathological remodeling, we stained the samples using Masson’s Trichrome staining. Unlike control group, we showed that the deposition of collagen fiber (blue-colored fibers) were increased in the periphery of bronchioles after the onset of chronic inflammation induced by OVA. Similar to the asthmatic rats, the levels of collagen fiber and pathological remodeling were not changed in the rats received c-kit^−^ cells. Of note, the injection of c-kit^+^ cells reduced intensity of collagen fibers and reached pathological changes comparable to the control group. IHC analysis revealed an increased VCAM-1 levels under asthmatic condition and the transplantation of c-kit^+^ cells blunted these effects. In contrast to c-kit^+^ cells, c-kit^−^ cells exerted less therapeutic effects to modulate VCAM-1 levels under asthmatic condition (Fig. 4).

**Table 2:** Pathological scores (for each group, *n* = 8). Statistical differences between control and different groups: + ; *p* < 0.05, +  + ; *p* < 0.01 and +  +  + ; *p* < 0.001. Statistical differences between CA + c-kit^+^ and CA + c-kit^−^ versus CA group: *; *p* < 0.05 and **; *p* < 0. 01. The lowest–highest pathological scores were showed in each experimental group between the parentheses

Pathological injuries	Scores in groups( for each group, *n* = 6)				
	(Minimum–Maximum)				
	C	CA	CA + c-kit^−^		CA + c-kit^+^
**Gablet cell proliferation**	(0–0)	(2–3) + + +		(2–3) + + +	(0–2) + *
**Interstitial pneumonia**	(0–0)	(1–3) + + +		(2–3) + + +	(1–2) + **
**Focal hemorragia**	(1–0)	(1–3) + + +		(1–3) + + +	(1–2) + + **
**Epithelial cells damage**	(0–0)	(2–3) + + +		(2–3) + +	(0–2) + **
**Atelectasis**	(0–0)	(1–3) + + +		(1–3) + +	(1–2) + *

### TNF-α and IL-1β contents were diminished in BALF after c-kit^+^ cell injection

**Fig. 5:**
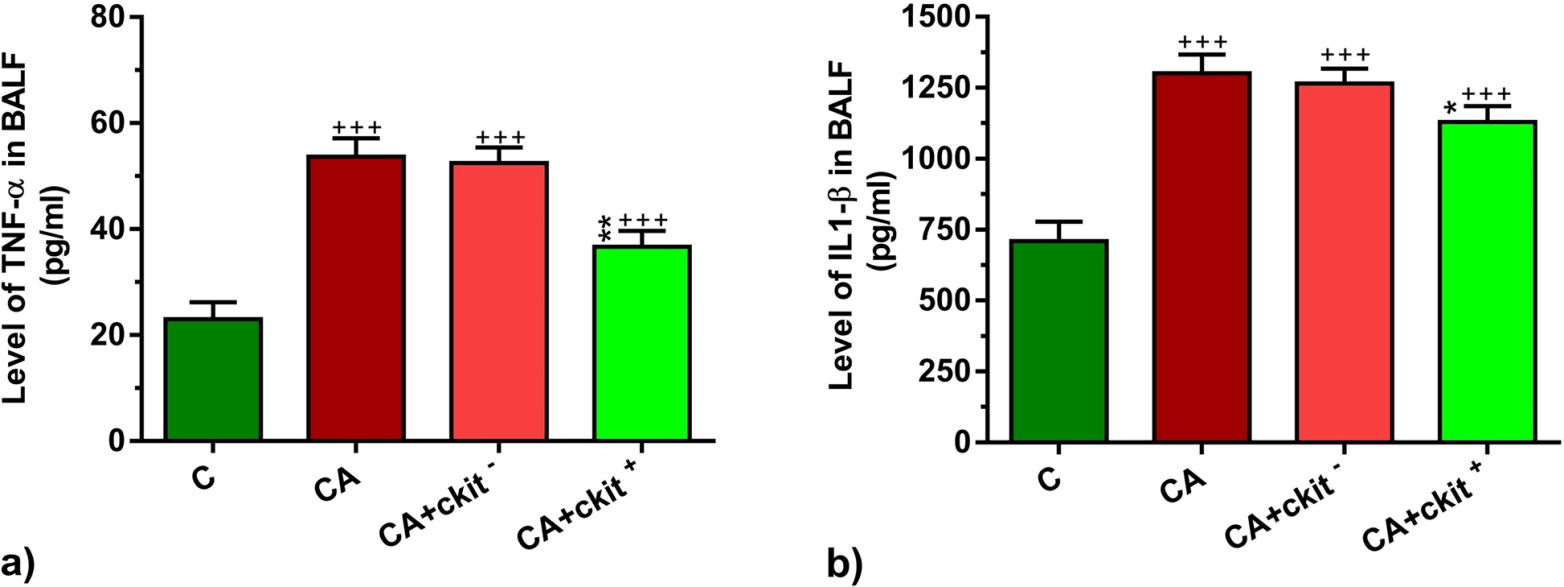
ELISA assay of BALF samples for the detection of TNF-α (**a**), IL-β (**b**) in C, CA, CA + c-kit^−^ CA + c-kit^+^ groups (*n* = 8). Statistical differences between control and different sensitized groups: +  +  + ; *p* < 0. 001. Statistical differences between CA + c-Kit^−^ and CA + c-Kit^+^ versus CA group: *; *p* < 0.05 and ***; *p* < 0. 001

Measuring BALF levels of TNF-α and IL-1β by ELISA exhibited significant changes in asthmatic animals related to the non-treated group (*p* < 0.001; Fig. 5a, b). We showed an inflammatory status in terms of cytokine levels like TNF-α and IL-1β after chronic sensitization. Of note, TNF-α and IL-1β were suppressed in the BALF of CA + c-Kit^+^ group as compared to the CA group (*p* < 0.01 to *p* < 0.05 respectively*;* Fig. 5a, b). As expected, c-kit^−^ cells cannot alter the cytokine levels 14 days after systemic injection. These data demonstrate that specific c-Kit subsets (c-Kit^+^ cells), but not c-kit^−^ cells, can cease the inflammation and pro-inflammatory cytokines after asthma induction.

### c-Kit + cells altered the expression of T helper-related cytokines and vascular adhesion molecules

**Fig. 6:**
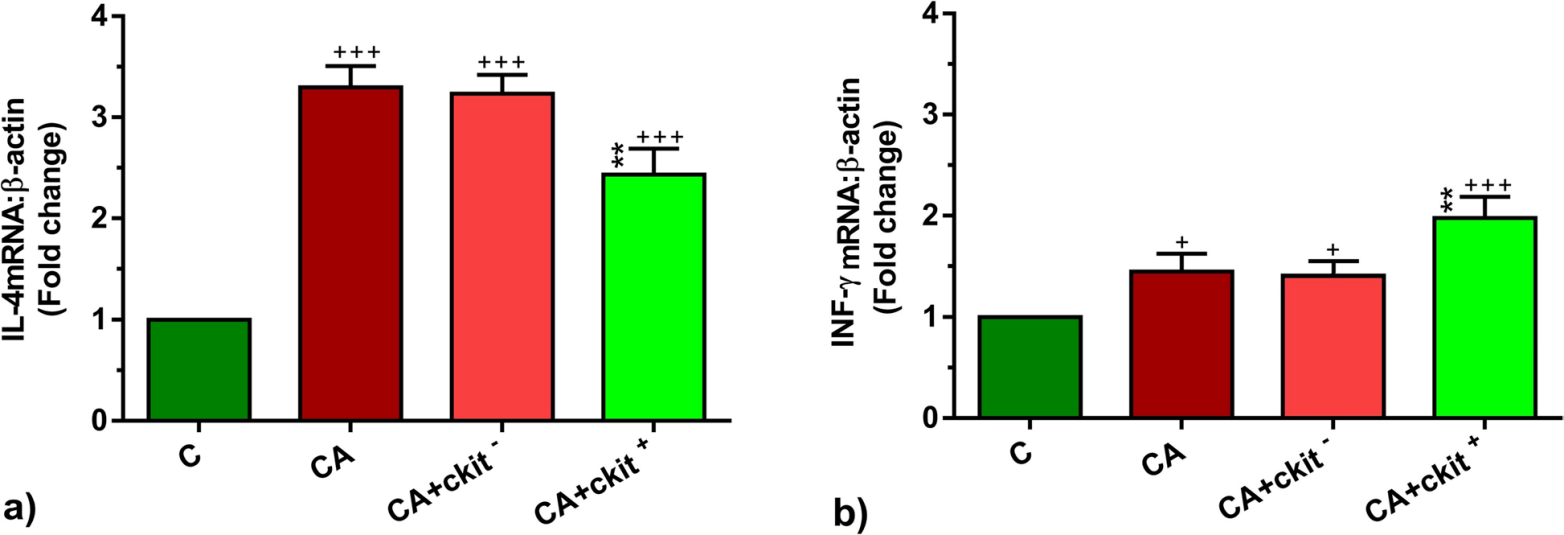
The levels of IL-4 (**a**) and INF-γ (**b**) mRNA expression in the lung tissues in C, CA, CA + c-kit^−^ CA + c-kit^+^ groups (*n* = 8). Statistical differences between control and different sensitized groups: + ; *p* < 0.05 and +  +  + ; *p* < 0. 001. Statistical differences between CA + c-Kit^−^ and CA + c-Kit^+^ versus CA group: **; *p* < 0. 01

**Fig. 7:**
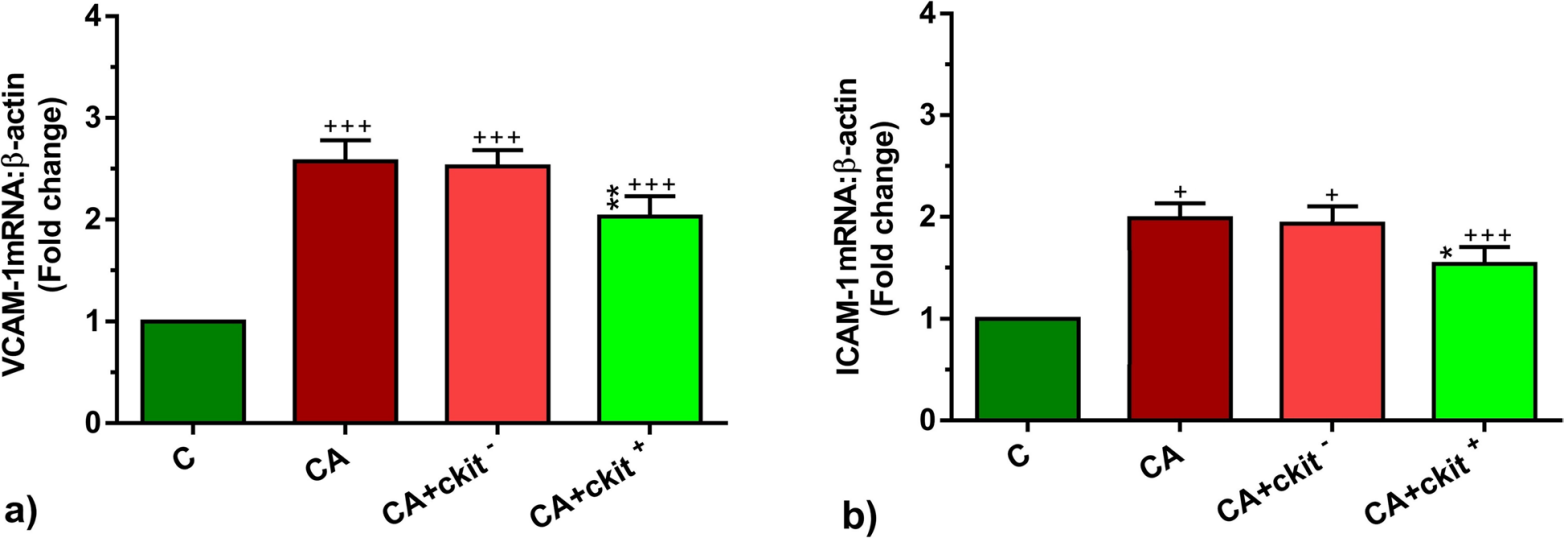
The levels of VCAM-1 (**a**) and VCAM-1 (**b**) mRNA expression in the lung tissues in C, CA, CA + c-kit^−^ CA + c-kit^+^ groups (*n* = 8). Statistical differences between control and different sensitized groups: +  +  + ; *p* < 0. 001. Statistical differences between CA + c-Kit^−^ and CA + c-Kit^+^ versus CA group: *; *p* < 0.05 and ***; *p* < 0. 001

To this end, IL-4 and INF-γ transcripts were significantly up-regulated in asthmatic rats related to the non-treated rats (*p* < 0.001 to *p* < 0.05 respectively*;* Fig. 6a, b). The transcription of IL-4 was reduced in the lung niche 14 days after c-Kit^+^ cells (*p* < 0.01; Fig. 6a). After c-Kit^+^ cell administration, we found accumulation of INF-γ even more than that of asthmatic condition (*p* < 0.01; Fig. 6b). C-kit^−^ cells did not alter the expression of above-mentioned cytokines compared to the asthmatic changes. Therefore, these data showed that c-Kit^+^ cells can alter the dynamic expression of IL-4 and INF-γ. Along with studying IL-4 and INF-γ levels, we also monitored the expression of endothelial adhesion molecules ICAM-1 and VCAM-1. Data showed enhanced ICAM-1 and VCAM-1 expression rate after onset of chronic asthmatic changes compared to the control group (*p* < 0.001; Fig. 7a, b). The injection of c-Kit^+^ cells, but not c-kit^−^ cells, can reduce the expression of ICAM-1 and VCAM-1 compared to the asthmatic group and reached to the near-to-control levels (*p* < 0.01 to *p* < 0.05 respectively*;* Fig. 7a, b). The reduction of ICAM-1 and VACAM-1 expression after c-Kit^+^ cell injection may relate to the regulated interaction of endothelial cells with inflammatory cells and blunted recruitment into the lungs.

### The levels of NO were reduced in asthmatic lungs after injection of c-Kit^+^ cells

**Fig. 8:**
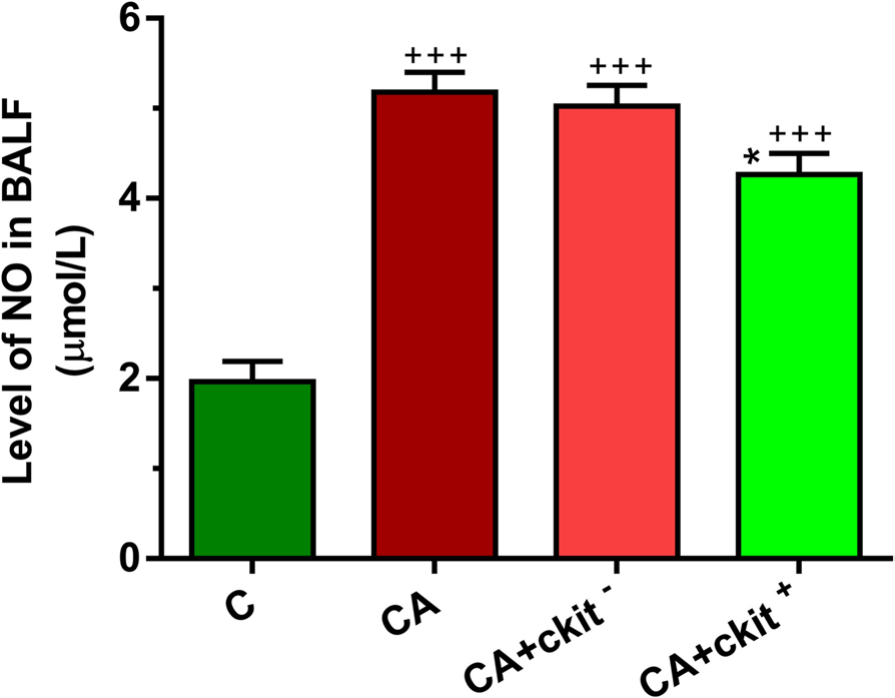
NO levels in BALF of in C, CA, CA + c-kit^−^ CA + c-kit^+^ groups (*n* = 8). Bars represent the mean ± SEM. Statistical differences between control and different sensitized groups: +  +  + ; *p* < 0. 001. Statistical differences between CA + c-Kit^−^ and CA + c-Kit^+^ versus CA group: *; *p* < 0.05

On this basis, we measured the levels of NO pre- and post- c-kit cell subsets. According to our data, the development of asthma was associated with the accumulation of NO inside the pulmonary tissue compared to the control group (*p* < 0.001; Fig. 8). We also found that the injection of c-kit^−^ cells did not alter the NO levels as compared to the asthmatic group and the NO levels were significantly elevated related to the control rats (*p* < 0.001; Fig. 8). The injection of c-kit^+^ cells did decrease the production of NO in asthmatic rats compared to the CA and CA + c-kit^−^ groups. However, the NO levels were higher as compared to the control healthy rats. Taken together, c-kit^+^ cells blunted nitrosative stress in asthmatic niche.

## Discussion

A plethora of accumulating data confirmed the regenerative significance of stem cell in healing of injured tissues which have no indication by conventional modalities such as asthma [7–9]. It was suggested this area opens new hopes in patients for prolonged survival rate and life expectancy. Up to date, both systemic and local administration routes are available for the transplantation of stem cells targeting specific pathologies like asthma [16, 17]. Noteworthy, each administration route has its own advantages/disadvantages with different therapeutic outcomes. Therefore, the selection of administration route should be done according to the status, extent and type of injury in the target tissue [12, 16, 18, 19].

To note, most of previously conducted experiments tried to examine the potency of several stem cell types during the asthmatic conditions in short-term asthma models which may not completely be comparable to the human asthma. To circumvent these pitfalls, attempts targeting to the development of chronic asthma are highly welcomed [3]. As a correlate, we tried to evaluate the systemic therapeutic effect of c-Kit^+^ cells in the model of rat with chronic asthma induced by 70-day procedure. Several subsets of stem cells with comparable therapeutic effects can be found in each tissue. Of these cells, c-Kit^+^ cells belonging to the progenitor cells have been used in the alleviation of different pathologies [12, 20]. As a separate note, the number of c-Kit^+^ cells are not enough in solid tissues such as lungs that hurdles up-to-the-minute application for several purposes. Besides, ethical issues and lack of donors limit the usual use of these cells [9, 12]. Bone marrow niche is as unique source for the isolation of different stem cell types with advantages compared to the other tissues [12, 21]. We noted systemic injection of c-Kit^+^ cells in chronic rat model of asthma can reduce pathological remodeling after 14 days. These features were determined with the alleviation of tissue injury. We found that the intensity of interstitial pneumonitis, focal hyperemia, atelectasis, fibrosis, bronchiolar epithelial cell injury and goblet cell proliferation were diminished in asthmatic rats received c-Kit^+^ cells. Monitoring the levels of IL-4 and INF-γ, TNF-α, IL-1β showed pro-inflammatory status in challenged rats after 70 days. Again, we found that c-Kit^+^ cells were eligible to modulate these features and closed near-to-control levels. Unlike, IL-4 and TNF-α, IL-1β cytokines, it was notified that INF-γ level was unexpectedly in asthmatic rats pre- and post-c-Kit^+^ cells. Due to the complexity of asthmatic condition, there are controversies related to the dynamic changes of INF-γ [2, 16, 22, 23]. In previous works conducted by our research group, we showed the decrease and increase of INF-γ after acute asthma induction and stem cell injection, respectively. By contrast, the levels of INF-γ were increased in CA and CA + c-Kit^+^ cells groups [2, 16]. To be specific, one reason would be that the severity of asthma and recruitment of type 2 T lymphocytes into the lung parenchyma can yield in paradoxical patterns associated with local INF-γ levels [23]. Here, we believe that the induction of chronic asthma model in rats in a 70-day period led to significant pathological outcomes which is supported by the up-regulation of vascular adhesion molecules like ICAM-1 and VCAM-1 involved in cell orientation from blood into the lung parenchyma. Additionally, because of chronic nature of asthma (as described in this study), this can give opportunity host tissue to compensate the pathological remodeling possibly via changing Th1/Th2 ratio [24]. As the injection of c-Kit^+^ cells reduced the expression of ICAM-1 and VCAM-1, it is noteworthy to mention that the recruitment of type 2 Th in latter phases is diminished which supported by data bright-filed images of CA + c-Kit^+^ cell group. Therefore, it should be noted that c-Kit^+^ cells homing is another logical reason for the elevation of INF-γ orchestrated via the alteration of resident immune cell juxtacrine and paracrine activity in lung parenchyma. It has been shown that the secretion of different soluble factors by c-Kit^+^ cells can blunt deleterious immune responses [12]. In addition, c-Kit^+^ cells did reduce the nitrosative stress and free radical inside asthmatic niche.

Commensurate with these comments, C-Kit^+^ cells can accelerate the healing procedure of chronic asthma via engaging different mechanisms. The up-regulation of adhesion molecules (ICAM-1, VCAM-1) and existence of bulk vascular niche help circulating C-Kit^+^ cells to redirect into the asthmatic parenchyma as indicated by IF imaging. In line with data driven from this study, the application of C-kit positive cells can exert therapeutic effects similarly in acute asthma model in the same species [12, 25].

This study faces some limitations. In crude enriched C-Kit^+^ cells, different subset exists. It is suggested to examine different subsets of C-Kit^+^ cells in rat model of chronic asthma. Whether a specific subset of C-Kit^+^ cells dominates after transplantation into the asthmatic niche is subject of attention and need more investigations. The possible interaction of transplanted exogenous C-Kit^+^ cells with local resident C-Kit^+^ cells should also be addressed for the prediction of therapeutic outcome. How C-Kit^+^ cells manage the immune cell recruitment into the asthmatic lungs after modulation of vascular adhesion surface molecules is mandatory to be monitored.

## Conclusion

This study indicated that systemically administrated c-kit + cells are effective in the reduction of chronic asthma-related pathologies via engaging several molecular mechanisms.

## Data Availability

The data to support the findings of this study are available from the corresponding author upon request.

## References

[1] Afzal S, Ramzan K, Waqar AB (2020). Alternative approaches for the treatment of Asthma and COPD: Focus on Cell-based therapies, Epigenetics, and Gene silencing approaches. Advancements in Life Sciences.

[2] Rahbarghazi R, Keyhanmanesh R, Aslani MR, Hassanpour M, Ahmadi M (2019). Bone marrow mesenchymal stem cells and condition media diminish inflammatory adhesion molecules of pulmonary endothelial cells in an ovalbumin-induced asthmatic rat model. Microvasc Res.

[3] Nials AT, Uddin S (2008). Mouse models of allergic asthma: acute and chronic allergen challenge. Dis Model Mech.

[4] Ahmadi M, Rahbarghazi R, Shahbazfar A-A, Keyhanmanesh R (2018). Monitoring IL-13 expression in relation to miRNA-155 and miRNA-133 changes following intra-tracheal administration of mesenchymal stem cells and conditioned media in ovalbumin-sensitized rats. The Thai Journal of Veterinary Medicine.

[5] McMillan S, Lloyd C (2004). Prolonged allergen challenge in mice leads to persistent airway remodelling. Clin Exp Allergy.

[6] Kumar RK, Foster PS (2002). Modeling allergic asthma in mice: pitfalls and opportunities. Am J Respir Cell Mol Biol.

[7] Cruz FF, Rocco PRM (2020). The potential of mesenchymal stem cell therapy for chronic lung disease. Expert Rev Respir Med.

[8] Mirershadi F, Ahmadi M, Rezabakhsh A, Rajabi H, Rahbarghazi R, Keyhanmanesh R (2020). Unraveling the therapeutic effects of mesenchymal stem cells in asthma. Stem Cell Res Ther.

[9] Rahbarghazi R, Kihanmanesh R, Rezaie J, Mirershadi F, Heiran H, Saghaei Bagheri H, Saberianpour S, Rezabakhsh A, Delkhosh A, Bagheri Y (2021). c-kit+ cells offer hopes in ameliorating asthmatic pathologies via regulation of miRNA-133 and-126. Iran J Basic Med Sci.

[10] Srour N, Thébaud B (2014). Stem cells in animal asthma models: a systematic review. Cytotherapy.

[11] Rolandsson Enes S, Weiss DJ (2020). Cell therapy for lung disease: current status and future prospects. Current Stem Cell Reports.

[12] Heiran H, Ahmadi M, Rahbarghazi R, Mir-ershadi F, Delkhosh A, Khaksar M, Heidarzadeh M, Keyhanmanesh R (2020). C-Kit+ progenitors restore rat asthmatic lung function by modulation of T-bet and GATA-3 expression. Exp Physiol.

[13] Yang Y-G, Tian W-M, Zhang H, Li M, Shang Y-X (2013). Nerve growth factor exacerbates allergic lung inflammation and airway remodeling in a rat model of chronic asthma. Exp Ther Med.

[14] Sheshpari S, Shahnazi M, Ahmadian S, Nouri M, Abbasi MM, Beheshti R, Rahbarghazi R, Honaramooz A, Mahdipour M (2020). Intra-Ovarian Injection of Bone Marrow c-Kit+ Cells Induced Ovarian Rejuvenation in Menopausal Rats.

[15] Zarafshan E, Rahbarghazi R, Rezaie J, Aslani MR, Saberianpour S, Ahmadi M, Keyhanmanesh R. Type 2 Diabetes Mellitus Provokes Rat Immune Cells Recruitment into the Pulmonary Niche by Up-regulation of Endothelial Adhesion Molecules. Adv Pharm Bull. 2022;12(1):176-82. 10.34172/apb.2022.019 PMC9012922 35517882

[16] Keyhanmanesh R, Rahbarghazi R, Aslani MR, Hassanpour M, Ahmadi M (2018). Systemic delivery of mesenchymal stem cells condition media in repeated doses acts as magic bullets in restoring IFN-γ/IL-4 balance in asthmatic rats. Life Sci.

[17] Tibboel J, Keijzer R, Reiss I, de Jongste JC, Post M (2014). Intravenous and intratracheal mesenchymal stromal cell injection in a mouse model of pulmonary emphysema. COPD.

[18] Abreu SC, Antunes MA, Maron-Gutierrez T, Cruz FF, Ornellas DS, Silva AL, Diaz BL, Ab'Saber AM, Capelozzi VL, Xisto DG (2013). Bone marrow mononuclear cell therapy in experimental allergic asthma: intratracheal versus intravenous administration. Respir Physiol Neurobiol.

[19] Trzil JE, Masseau I, Webb TL, Chang CH, Dodam JR, Cohn LA, Liu H, Quimby JM, Dow SW, Reinero CR (2014). Long-term evaluation of mesenchymal stem cell therapy in a feline model of chronic allergic asthma. Clin Exp Allergy.

[20] Marino F, Scalise M, Cianflone E, Mancuso T, Aquila I, Agosti V, Torella M, Paolino D, Mollace V, Nadal-Ginard B (2019). Role of c-kit in myocardial regeneration and aging. Front Endocrinol.

[21] Astori G, Soncin S, Cicero VL, Siclari F, Sürder D, Turchetto L, Soldati G, Moccetti T (2010). Bone marrow derived stem cells in regenerative medicine as advanced therapy medicinal products. American journal of translational research.

[22] Balkrishna A, Solleti SK, Singh H, Tomer M, Sharma N, Varshney A (2020). Calcio-herbal formulation, Divya-Swasari-Ras, alleviates chronic inflammation and suppresses airway remodelling in mouse model of allergic asthma by modulating pro-inflammatory cytokine response. Biomedicine & Pharmacotherapy.

[23] Lin L-J, Huang HY (2020). DFSG, a novel herbal cocktail with anti-asthma activity, suppressed MUC5AC in A549 cells and alleviated allergic airway hypersensitivity and inflammatory cell infiltration in a chronic asthma mouse model. Biomedicine & Pharmacotherapy.

[24] Biller H, Bade B, Matthys H, Luttmann W, Virchow J (2001). Interferon-g secretion of peripheral blood CD81 T lymphocytes in patients with bronchial asthma: In vitro stimulus determines cytokine production. Clin ExpImmunol.

[25] Mirershadi F, Ahmadi M, Rahbarghazi R, Heiran H, Keyhanmanesh R (2021). C-Kit+ Cells Can Modulate Asthmatic Condition via Differentiation Into Pneumocyte-Like Cells and Alteration of Inflammatory Responses via ERK/NF-κB Pathway.

